# SyntDB: defining orthologues of human long noncoding RNAs across primates

**DOI:** 10.1093/nar/gkz941

**Published:** 2019-11-15

**Authors:** Oleksii Bryzghalov, Michał Wojciech Szcześniak, Izabela Makałowska

**Affiliations:** Adam Mickiewicz University in Poznan, Faculty of Biology, Institute of Anthropology, Laboratory of Integrative Genomics, Uniwersytetu Poznańskiego 6, 61-614 Poznan, Poland

## Abstract

SyntDB (http://syntdb.amu.edu.pl/) is a collection of data on long noncoding RNAs (lncRNAs) and their evolutionary relationships in twelve primate species, including humans. This is the first database dedicated to primate lncRNAs, thousands of which are uniquely stored in SyntDB. The lncRNAs were predicted with our computational pipeline using publicly available RNA-Seq data spanning diverse tissues and organs. Most of the species included in SyntDB still lack lncRNA annotations in public resources. In addition to providing users with unique sets of lncRNAs and their characteristics, SyntDB provides data on orthology relationships between the lncRNAs of humans and other primates, which are not available on this scale elsewhere. Keeping in mind that only a small fraction of currently known human lncRNAs have been functionally characterized and that lncRNA conservation is frequently used to identify the most relevant lncRNAs for functional studies, we believe that SyntDB will contribute to ongoing research aimed at deciphering the biological roles of lncRNAs.

## INTRODUCTION

Long non-coding RNAs (lncRNAs) represent a highly heterogeneous class of RNA molecules arbitrarily defined as transcripts of more than 200 nucleotides in length that are not translated into proteins. A rapidly growing number of studies highlight their essential biological roles in processes such as transcription, splicing, translation, the cell cycle and apoptosis, protein localization, imprinting or stem cell pluripotency (1). They have also been implicated in human diseases; e.g., lncRNAs have been linked to malignant transformation, and a number of them represent diagnostic and prognostic biomarkers for cancers (2). lncRNAs play these roles in different ways, including through direct RNA:RNA interactions, miRNA sponge activity, nucleosome repositioning, histone modifications, DNA methylation or binding the transport factors to inhibit the nuclear localization of specific transcription factors (3,4). This heterogeneity in the modes of action and roles of lncRNA molecules poses a major challenge in functional studies of lncRNAs, such that only a small fraction of them are well characterized. This issue can be partially mitigated by the analysis of conservation in two ways. First, the level of conservation indicates whether a given lncRNA is most probably functional or represents mere transcriptional noise. However, one should keep in mind that there are plentiful functional, non-conserved lncRNAs, including human-specific lncRNAs, such as MSTRG.141391.4, an isoform of p53-induced noncoding RNA (PINCR), which plays a critical prosurvival role in the response to DNA damage (5). Second, knowledge of the level of lncRNA conservation and lncRNA orthologues helps to assign lncRNAs to one of the hypothetical functional domains. In particular, exon-level conservation is a strong indicator of functionalities mediated by mature RNA sequences such as lncRNA:RNA interactions involved in the regulation of RNA processing, stability and expression (6,7). However, a growing body of evidence shows that for most human lncRNAs, their transcription alone, rather than the production of mature RNA molecules, is of biological importance. For example, up to 70% of human genes show evidence of antisense transcription giving rise to so-called natural antisense transcripts (NATs) (8,9), whose function is to regulate the expression of their sense partners. This can be achieved in multiple ways (10,11), but the prevailing mechanism is the recruitment of complex epigenetic machinery that mediates histone modifications, leading to transcriptional deregulation of target genes (12). The underlying RNA:protein interactions involve little or no sequence specificity; hence, virtually no constraints upon primary sequence conservation exist. Such lncRNAs typically display either locus-level conservation, with splicing patterns not being preserved across orthologues, or only conservation of their genomic location relative to protein-coding genes, presumably being under their control (13,14).

Keeping in mind the very limited availability of orthologous relationships for human lncRNAs and the importance of such data in functional studies, we searched for lncRNA orthologues across eleven primate species and classified them into those showing exonic identity, locus identity or only positional conservation (syntologs) (15). The analyses were performed using our custom pipeline built upon the *slncky* tool (15), used for the detection and prioritization of lncRNA orthologues found within custom lncRNA annotations that we obtained from vast publicly available RNA-Seq data sets. The resulting unique sets of lncRNAs for eleven primates and the identified orthologues with accompanying data are made available in the newly developed online database SyntDB (http://syntdb.amu.edu.pl/). This resource stores over 78 000 expressed human lncRNAs and 2054 to 18 226 lncRNAs for each of the other primate species. The uniqueness of the stored data and the modern user interface with a number of browse, search and on-the-fly visualization options, full data download capability and transparency, and visual summaries for each of the primate transcriptomes make SyntDB a user-friendly and potentially useful resource for ongoing lncRNA research.

## MATERIALS AND METHODS

### *Ab initio* transcriptome assembly

For each of the non-human primate species, RNA-Seq data were downloaded from the Sequence Read Archive (16) (Supplementary File 1) and were subjected to quality trimming and adapter clipping with Trimmomatic using the default settings except for *LEADING:20*, *TRAILING:20*, *SLIDINGWINDOW:5:20* and *MINLEN:50*. To remove rRNA-derived reads, mapping against a set of ribosomal RNAs was performed with Bowtie 2 (17), and only unmapped reads were retained. Ribosomal RNAs from humans and the primate species of interest were retrieved from ENSEMBL (18) and/or NCBI’s RefSeq (19) based on availability (Supplementary File 2). The clean reads were then mapped against the corresponding primate reference genome (Supplementary File 2) with HISAT (20). Here, GTF files from ENSEMBL or NCBI were used as a reference to further improve the performance of the program. The resulting SAM files, one per sample, were converted into the BAM format and sorted by coordinates with SAMtools (21). The BAM files were then applied for *ab initio* transcriptome assembly and quantification with StringTie v1.3.3b (22), again using species-specific GTF files as a reference. This produced new GTF files with custom transcriptomes, one per sample. The transcriptomes were subjected to filtering to retain only the most credible transcripts. First, an expression filter was applied to retain only the transcripts expressed at a minimum of 1 transcript per million (TPM). Then, the transcriptomes were compared against reference annotations with Cuffcompare (v2.2.1) from the Cufflinks package (23), and transcripts belonging to one of the following class codes were removed, as they represent potential errors in transcriptome assembly: c, e, p and s (the meaning of the class codes is provided in Supplementary File 3). Finally, the *ab initio* assemblies were merged into a single transcriptome per species in a GTF format using Cuffmerge from the Cufflinks package.

### Identification of lncRNAs

Transcript sequences in FASTA format were extracted from the corresponding genome based on the GTF file data with the assembled transcriptome. The same GTF file was compared against reference annotations using Cuffcompare (v2.2.1) from the Cufflinks package with the -R (considering only the reference transcripts that overlap any of the input transfrags) and -C (including the ‘contained’ transcripts in the .combined.gtf file) options. Then, lncRNA identification was performed with the following settings, as implemented in in-house Python scripts:

- Transcripts with Cuffcompare class codes *=* , *j*, *o* (Supplementary File 3) were discarded if a reference gene was not classified as a lncRNA in ENSEMBL. In the case of species that were not annotated in ENSEMBL (*Chlorocebus sabaeus*, *Macaca fascicularis*, *Pan paniscus*, *Cercocebus atys*), a BLASTN (24) search against the RFAM database (25) was performed, and based on an E-value threshold of 1e-10, sequences with hits to the following classes of RNAs were discarded: tRNA, snRNA, snoRNA, rRNA, miRNA and scaRNA.
- Transcripts shorter than 200 bases were removed.
- Transcripts containing open reading frames (ORFs) identified using TransDecoder v5.0.2 (26) with the -m 100 (minimum protein length; default: 100) and -S (*strand-specific*) options were discarded.
- Transcripts classified as *coding* by the Coding Potential Calculator (CPC, version 0.9-r2) (27) with default settings were eliminated.

Regardless of the TransDecoder and CPC results, we retained all expressed RNAs classified as lncRNAs in ENSEMBL. For humans, the lncRNAs and the associated data such as expression levels and tissue specificity *Tau* scores came from our recent study (28).

### Identification of lncRNA orthologs

#### Preparation of input data

**Figure 1.**
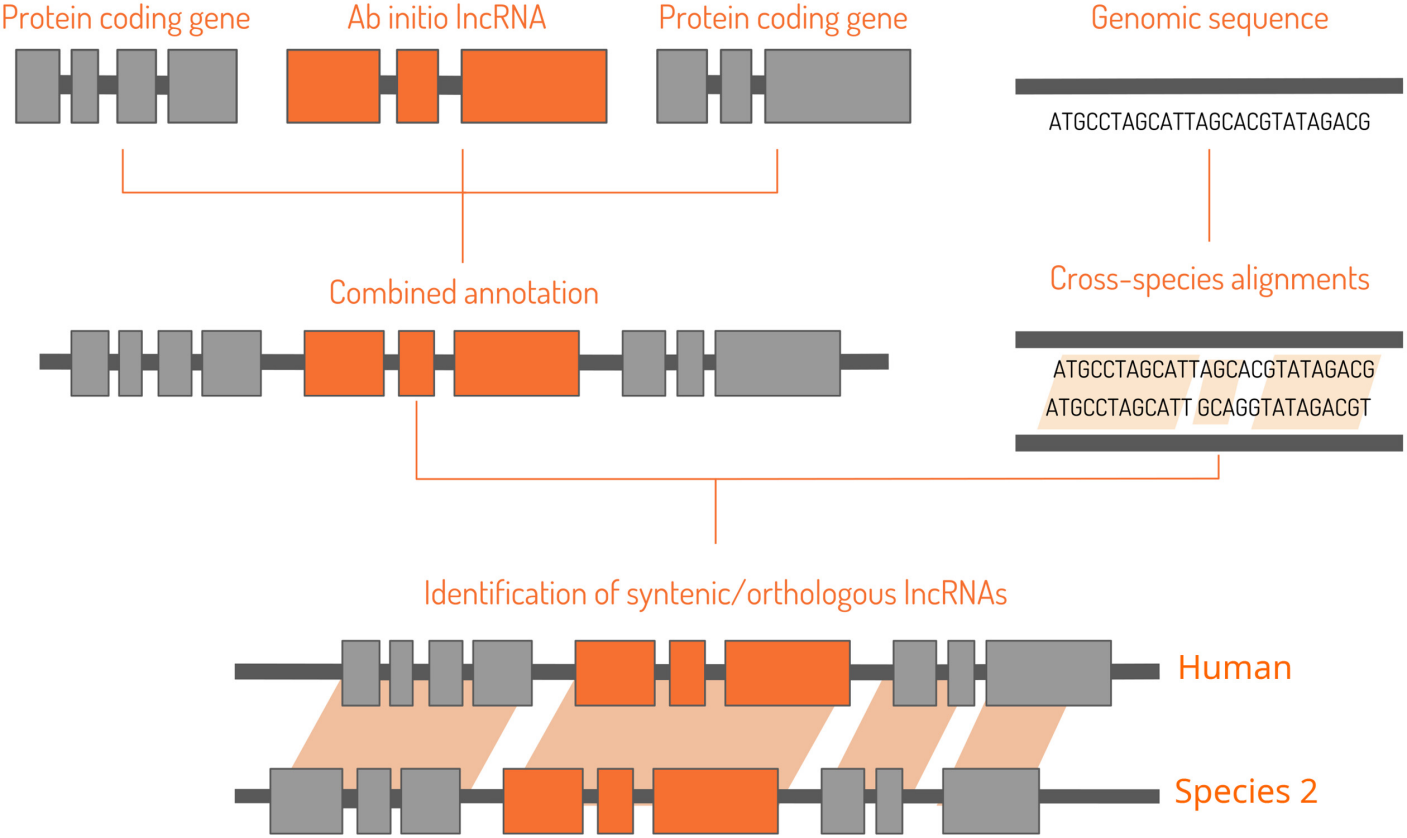
Schematic representation of the orthologues search procedure. In the first step, custom genome annotations are built using our sets of predicted lncRNAs and annotations from ENSEMBL or NCBI. To enable direct species-to-species comparisons, whole-genome alignments are generated. With these input data, syntenic regions are identified with liftOver, then aligned with reduced gap penalties using lastz, filtered and prioritized via statistical data analysis, and pairs of conserved lncRNAs are finally reported.

The primate annotations comprised two components: our lncRNA predictions and annotations from NCBI or ENSEMBL (Figure 1). ENSEMBL served as a source of protein-coding, miRNA and snoRNA gene annotations for most primates, except for *Pan paniscus*, *Cercocebus atys*, *Macaca fascicularis* and *Chlorocebus sabaeus*. To generate chain files with pairwise genome alignments, we applied whole-genome alignment tools (https://github.com/ge11232002/CSC/tree/master/WholeGenomeAlignment/pipelines). As an input, we used 2bit files produced from FASTA genome files using the faToTwoBit command-line utility (https://github.com/ENCODE-DCC/kentUtils). Here, the human 2bit file was used as a target, while the given primate 2bit genome served as a query.

#### Implementation of the core algorithm

As a core of the orthologous search pipeline, we used *slncky* (15), a sensitive method for orthologous lncRNA alignment (Figure 1). In the search for conserved lncRNA orthologs, *slncky* defines the syntenic regions of two genomes with *liftOver* (15). Based on the recommendations provided by the liftOver package manual, we used the *-minMatch* parameter with a value of 0.01 (https://github.com/ENCODE-DCC/kentUtils/tree/master/src/hg/liftOver). If a noncoding transcript exists in the syntenic region, *slncky* aligns the area from 100 000 base pairs upstream to 100 000 base pairs downstream of the two syntenic regions. We decided to use the *–pad 100,000* parameter value because in comparison with the default value of 50 000, it is more likely to contain an alignable coding transcript, either up- or downstream of the lncRNA, which in turn helps *lastz* (https://github.com/lastz/lastz) to find a positively scoring alignment. We also checked the option of 150 000 bases, as it was recommended in the original paper (15), and significant differences were not reported. We retained the default value for the reduced gap-opening penalty (*−−gap  =  25 040*) because of many small insertions that appear to be well tolerated by lncRNA transcripts, as proposed by the tool's developers. Finally, to reduce reporting alignments that may be driven by repetitive elements, *slncky* aligns each lncRNA to the shuffled intergenic regions and seeks to establish a null distribution and to determine the empirical 5% threshold for significant alignment scores.

### Database construction

The database represents a LAMP stack (https://hub.docker.com/r/mattrayner/lamp), containerized with docker and constructed with PHP (7.3.6), Apache (v. 2.4.29) and mySQL (v. 5.7.26). To build a user interface, we used the Bootstrap framework (https://getbootstrap.com/). Selected charts are created in Python using the Plotly (https://plot.ly/), Matplotlib (https://matplotlib.org/), Seaborn (https://seaborn.pydata.org/) and Pygal (http://pygal.org/en/stable/) libraries, whereas the ChartJS framework of JavaScript (https://www.chartjs.org/) is utilized for the vast majority of charts. The interface is optimized for the Firefox (68.0.1 and higher) and Chromium (v75 and higher) browsers. To decrease the access time, we used a content distribution network for the Google font API, Bootstrap CSS, animate.css (https://github.com/daneden/animate.css/) and chartJS. We also used gzip compression for transferring compressible responses and images. The performance of SyntDB was checked using WebPageTest (www.webpagetest.org), with the only issue being a slightly elevated First Byte Time parameter (up to ∼1.5 s) for selected pages (e.g. lnc_example.php) that execute multiple requests to a mySQL server.

## RESULTS AND DISCUSSION

### Summary of the stored data and comparison with similar databases

**Figure 2.**
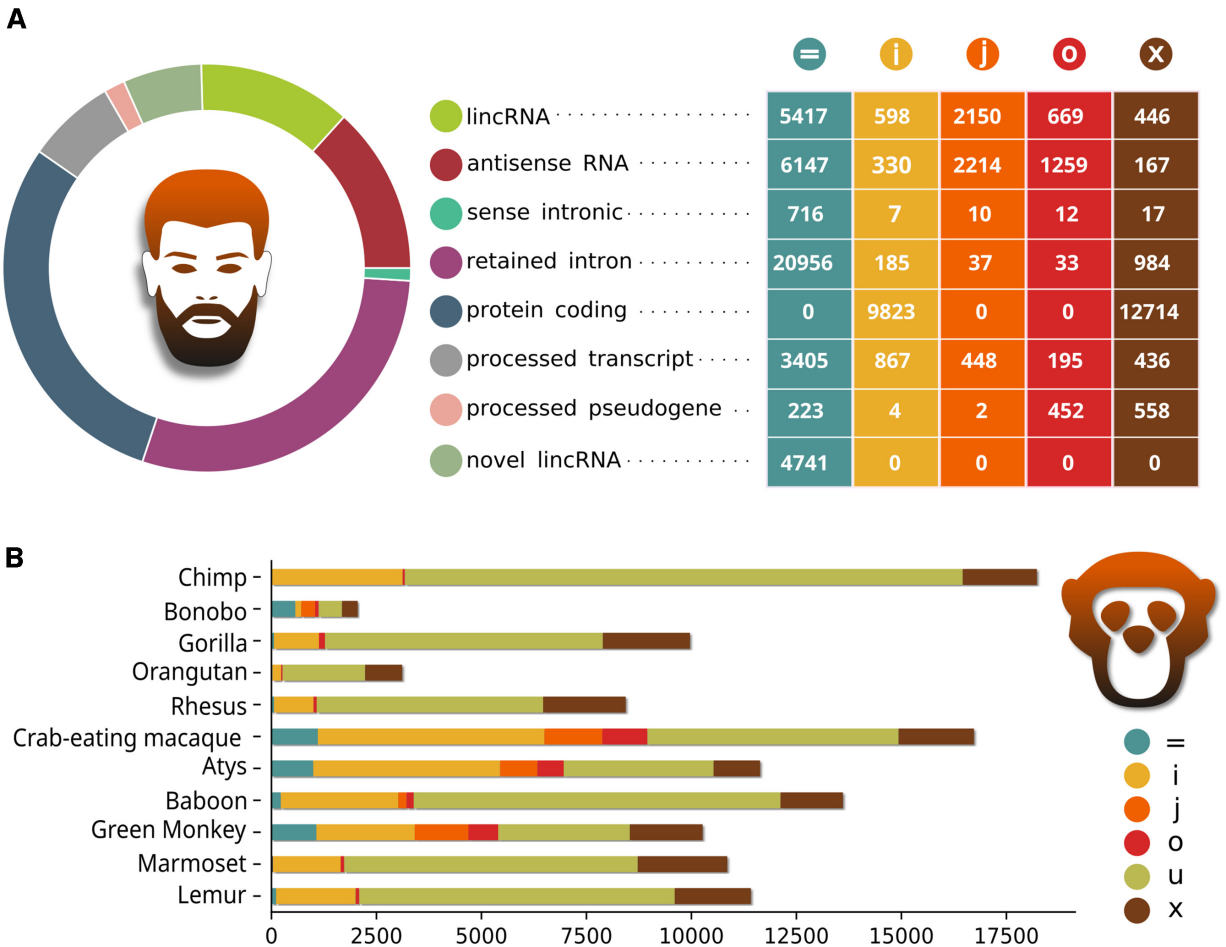
Characteristics of human (**A**) and non-human (**B**) sets of lncRNAs. The lncRNAs were compared with Cuffcompare against reference annotations for a given species, producing class codes that show relationships between a given lncRNA and the closest reference transcript. ‘Novel lincRNAs’ represent new lncRNA loci, while the other transcript types, such as the ‘protein coding’ and ‘retained intron’ categories, refer to the closest reference transcript and were assigned by ENSEMBL. For example, in the case of the ‘protein coding’ type, the lncRNA is either antisense to a protein-coding gene or is expressed in its intron (class codes ‘x’ or ‘i’). Class codes legend: ‘ = ’ a complete match of an intron chain; ‘i’ a transfrag falling entirely within a reference intron; ‘j’ a potentially novel isoform; ‘o’ a generic exonic overlap with a reference transcript; ‘x’ an exonic overlap with a reference on the opposite strand; ‘’u’ unknown, intergenic transcript (representing a novel lincRNA).

We previously compiled a set of 78 514 human lncRNAs using RNA-Seq data from 1,463 samples (28) available in ENCODE (29), which served as a reference catalogue of human lncRNAs in the conservation study. The dataset contains 41 855 lncRNAs identical to ENSEMBL transcripts, 4741 lncRNAs belonging to previously unannotated genomic loci and 37 114 transcripts that overlap with ENSEMBL genes, including novel splicing isoforms or antisense lncRNAs (Figure 2). The newly assembled noncoding transcriptomes of the other primates varied in size from 2055 lncRNAs in bonobo to 18 226 in chimpanzee (Supplementary File 2). Although these two species are closely related, this difference stems from the amount of available RNA-Seq data and, consequently, the quality of the obtained transcriptome assemblies. Only a moderate fraction of the lncRNAs are identical to those stored in ENSEMBL, and the majority of them represent new intronic lncRNAs, new antisense lncRNAs or transcripts of newly discovered lncRNA loci (new lincRNAs). Overall, SyntDB stores 78 514 human lncRNAs and 137 897 lncRNAs identified in the other eleven primate species. There is no other publicly available data repository of primate lncRNAs on this scale. For example, NONCODE (30) stores lncRNAs found in four primates: chimp, gorilla, orangutan and rhesus, while Deepbase 2.0 (31) includes only chimp, gorilla and rhesus. Both NONCODE and Deepbase provide conservation data for human lncRNAs. To identify conserved counterparts between species, NONCODE utilizes UCSC LiftOver. However, this feature is limited to the four species mentioned above. Deepbase 2.0 instead resolves orthologous relationships using BLASTN and reports transcripts with the lowest E-values, thus disregarding the fact that many conserved lncRNAs represent positionally conserved syntologs with no detectable sequence similarity between species.

### Overview of SyntDB functionalities

The navigation menu provides direct access to the main search page, a page with an example human lncRNA, and *Data* menu items. The latter is divided into 4 parts: (i) conservation data summaries; (ii) human transcriptome summaries; (iii) summaries for non-human primate transcriptomes and (iv) a section with a number of options to download datasets in a CSV format as well as a full dump of the database.

SyntDB allows searches across reference human lncRNAs using common IDs such as ENSEMBL transcript/gene IDs or a gene name. One can also perform a sequence-based search with BLASTN. When searching by IDs or gene names, it is not only transcripts identical to ENSEMBL annotations that are returned but also any lncRNAs that are related to a gene or transcript of interest. For instance, the lncRNA MSTRG.6478.2 is transcribed in antisense to the human protein-coding gene NBPF26 (ENSG00000273136); thus, a search by ENSG00000273136 or NBPF26 returns the MSTRG.6478.2 lncRNA among the displayed results. Detailed information about the possible relationships of lncRNAs with reference ENSEMBL genes based on the original Cuffcompare class codes is provided in Supplementary File 3. After the search is performed, a list of lncRNA transcripts that meet the user's criteria is displayed. For each human noncoding transcript, there are three tabs: *Human transcript*, *Conserved counterparts*, and *Browse the genome region*. The first tab provides detailed information on an lncRNA, such as its genomic localization, expression, a table with the other splicing isoforms of a given lncRNA gene, ‘Disease and interactions’ section with experimentally validated data from LIVE (32) and EVLncRNAs (33) databases (if available), FASTA sequence and a brief conservation overview. The second tab is dedicated to orthologues found across primates, presented as a dendrogram with exonic and locus identities across primates as well as links to detailed data on the two conserved counterparts. Finally, the *Browse the genome region* tab is designed to integrate the human lncRNA transcripts with external resources. Here, we implemented the Biodalliance genome browser (https://www.biodalliance.org/) with our lncRNAs as well as GENCODE (34) annotations and the phastCons46way conservation track (35). Additionally, links to the ENSEMBL and UCSC (36) genome browsers are available there.

### lncRNA conservation data in SyntDB

**Figure 3.**
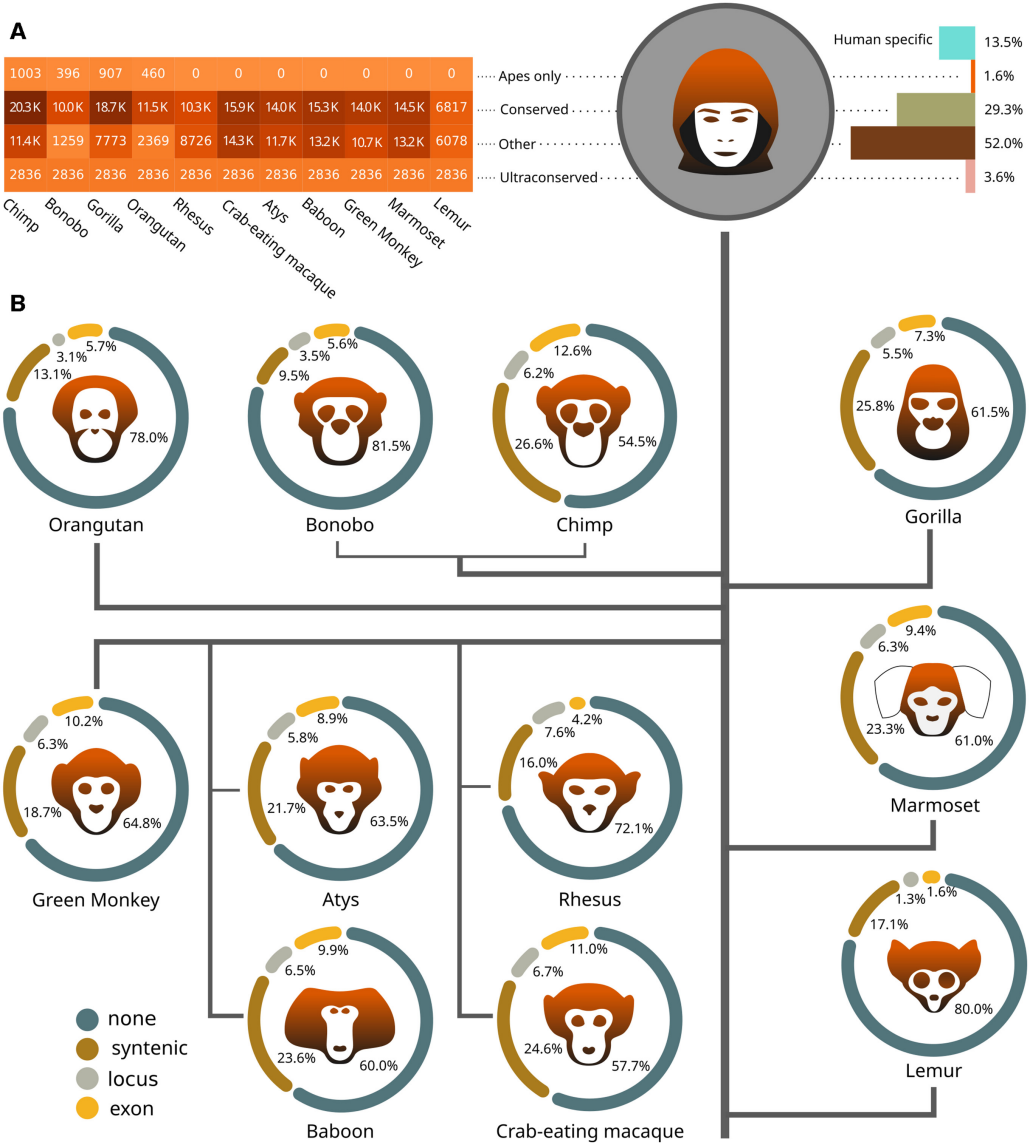
(**A**) The table presents numbers of human lncRNAs with orthologues found in particular primate species, classified into groups based on the depth of evolutionary conservation: ape-specific, conserved and ultraconserved lncRNAs, as described in the main text. The counts of lncRNAs that failed to be classified (designated other) are also provided, such as lncRNAs for which orthologues were only found in a single species. (**B**) Distribution of orthology evidence types across primates. Syntenic: lncRNAs showing only positional conservation (syntologs); locus: orthologues identified based on locus sequence similarity, with no splicing pattern preserved; exon: orthologues identified based on exon sequence similarity; none: human lncRNAs with no conserved counterparts in a given species.

Evolutionary conservation often plays a key role in pinpointing genes playing crucial biological roles; however, the dynamics of lncRNA sequence evolution make this task much more demanding than in the case of protein-coding genes. It has been proven that not only do the exons of lncRNA sequences evolve faster than those located in protein-coding genes (37), but lncRNA exons also display only modestly higher levels of evolutionary conservation than their introns. Nevertheless, in the context of lncRNAs, a lack of sequence or secondary structure conservation does not imply non-functionality (38). Indeed, there are human- or primate-specific lncRNAs that have been proven to be functional or therapeutically relevant (39). On the other hand, there are a number of evolutionarily conserved lncRNAs that play critical roles in cells, such as MALAT, XIST or HOTAIR, which have orthologues in SyntDB. Researchers often select lncRNAs for in-depth studies based on their conservation. Keeping this in mind, we prepared four sets of human lncRNAs based on the depth of their conservation (Figure 3): (i) *human-specific* lncRNAs, (ii) *great-ape specific* lncRNAs whose orthologues were identified in at least two of the following great apes species and no others: *Pan troglodytes*, *Pan paniscus*, *Pongo abelii* and *Gorilla gorilla*, (iii) *conserved* lncRNAs with orthologues found in great apes and other species and (iv) *ultraconserved* lncRNAs identified in all eleven primate species. Interestingly, only 20.97% of the *great ape-specific* lncRNAs have orthologues showing exonic identity, but this fraction rises to 28.18% in the case of *conserved* lncRNAs and as high as 42.86% for the *ultraconserved* lncRNAs. A reverse trend can be seen for fractions of lncRNAs showing only positional conservation (syntologs), which reaches 66.78% in the case of *great-ape-specific* lncRNAs, 55.51% in the *conserved* set and only 42.37% in *ultraconserved* lncRNAs. These data show that the most conserved lncRNAs evolve under stronger constraints on their mature RNA sequence, and they are therefore expected to play biological roles where mature lncRNA products are important more often.

Once orthologous lncRNAs are identified, one would typically ask the question of whether the two lncRNAs are functional and play similar biological roles. A very crude proxy could be given by the analysis of expression. It has been demonstrated that the tissue specificity and expression patterns of functional genes are generally conserved across species (13). According to our data, the tissue specificity of human lncRNAs strongly correlates with lncRNA conservation, with *Tau* scores for *human-specific* lncRNAs that are significantly higher than in any other group (Mann-Whitney, *P* < 1e−6). This finding is in line with the fact that biologically significant genes, including many protein-coding genes, tend to be more broadly expressed than evolutionary novelties with no established function (40). We also found that the most conserved lncRNAs (those belonging to *conserved* and *ultraconserved* groups) displayed significantly higher expression levels than the *human-specific* lncRNAs (Mann−Whitney, *P* = 3.43e−12 and 1.64e−13, respectively). Detailed expression data for particular human lncRNAs and their orthologues in primates are available in SyntDB.

A key advantage of SyntDB is that it provides conserved counterparts for human lncRNAs, including those with no detectable exon-level conservation. Such conserved pairs of lncRNAs could be divided into two categories: those showing locus-sequence identity (with no splice site conservation) and those whose expression is tied to homologous genomic loci but for which sequence similarity was not detected (syntologs). Our approach for identifying such cases relies on whole-genome alignments (WGA), which project expressed lncRNA loci to the corresponding loci in the other species. Notably, it heavily relies on genome annotations in two ways. First, conserved, neighbouring protein-coding genes are used to resolve spurious conservation relationships. Second, the ability to efficiently detect lncRNA orthologues requires deep annotations of lncRNAs in a given species, which we prepared using a bulk of available RNA-Seq data from the Sequence Read Archive database. Custom WGAs as well as enhanced genome annotations with relatively rich sets of expressed lncRNAs are key advantages of our methodology. It is also noteworthy that the strength of evidence for positional conservation depends on the evolutionary distance between the species of interest. We mitigate this limitation by using closely related primate species. We are aware, however, that our orthology inference pipeline fails to unanimously pinpoint lncRNA homologs in cases where more than one lncRNA can be found at neighbouring genomic loci or in the neighbourhood of protein-coding genes. In such cases, a single orthologous pair is selected based on statistical analysis but with no guarantee that the best solution is provided.

## DATA AVAILABILITY

SyntDB is free and publicly available at http://syntdb.amu.edu.pl/; full database dump as well as specific data downloads are enabled.

## Supplementary Material

gkz941_Supplemental_File
