# Supplementary material for: SyntDB: defining orthologues of human long noncoding RNAs across primates
Source: Nucleic Acids Res. 2019 Nov 15;48(D1):D238–45. doi: 10.1093/nar/gkz941 (PMC7145678; doi:10.1093/nar/gkz941)
Supplement: gkz941_Supplemental_File [file gkz941_supplemental_file.pdf]

Suppl. File 1
A list of RNA-Seq libraries used in this research for *ab initio* transcriptome assembly in eleven primate species.

Species	Run accession	Source
	SRR629519	Unspecified
	SRR629515	Unspecified
	ERR731511	Unspecified
	SRR1758988	Spleen
	SRR1758987	Skeletal muscle
	SRR1758986	Lymph node
	SRR1758985	Lung
	SRR1758984	Liver
	SRR1758983	Kidney
	SRR1758982	Heart
	SRR1758980	Colon
	SRR1758979	Brain right hemisphere
	SRR1758978	Brain pituitary
	SRR1758977	Brain left hemisphere
	SRR606715	testis tissue
	SRR952610	Generic sample
	SRR867044	Cerebellum tissue
	SRR867043	Cerebral cortex tissue
	SRR866209	Hippocampus tissue
**Callithrix jacchus**	SRR866208	Bladder tissue
	SRR1759003	Spleen
	SRR1759002	Spleen
	SRR1759001	Skeletal muscle
	SRR1759000	Skeletal muscle
	SRR1758999	Skeletal muscle
	SRR1758998	Lung
	SRR1758997	Liver
	SRR1758996	Kidney
	SRR1758995	Kidney
	SRR1758994	Colon
	SRR832933	Unspecified
	SRR1758989	Cerebellum
	SRR1758990	Cerebellum
	SRR1758991	Frontal cortex
**Microcebus**	SRR1758992	Frontal cortex
**murinus**	SRR1758993	Temporal Lobe
	SRR1778441	spleen
	SRR1746861	Hippocampus (brain)
	SRR1602582	blood
	SRR1575130	Pluripotent Stem Cells
	SRR1369674	Bone Marrow
	SRR1282481	PBMC

	SRR1282462	unspecifed
	SRR1240160	rectal mucosa
	SRR1048277	embryonic stem cells
	SRR594462	spleen
	SRR594460	lung
	SRR594458	kidney
	SRR389102	skeletal muscle
	SRR389099	adipose
	SRR223522	quadriceps
	ERR247244	unspecifed
**Macaca mulatta**	SRR389100	prefrontal cortex
	SRR1636484	Hippocampus (brain)
	SRR1636420	blood
	SRR1636398	Fibroblast
	SRR1636374	Pituitary
	SRR1636350	Adrenal
	DRR018834	Vero cell line JCRB0111, kidney epithelium
	SRR1184473	Adrenal
	SRR1178505	Pituitary gland
	SRR1032472	Fibroblasts
	SRR1032471	Caudate
	SRR1032466	Pituitarygland
	SRR1032454	Blood
	SRR1032365	Caudate
	SRR1032364	Blood
	SRR1032363	Pituitarygland
	SRR1032370	Fibroblast
	SRR1178508	Adrenal
	SRR1636448	Hippocampus (brain)
	SRR1636489	Hippocampus (brain)
**Chlorocebus sabaeus**	SRR1636445	Caudate
	SRR1758933	Thymus
	SRR1758932	Spleen
	SRR1758931	Spleen
	SRR1758930	Skeletal muscle
	SRR1758929	Skeletal muscle
	SRR1758928	Lymph node
	SRR1758927	Lung
	SRR1758926	Lung
	SRR1758925	liver
	SRR1758924	Liver
	SRR1758923	Kidney
	SRR1758922	Kidney

	SRR1758920	Colon
	SRR1758919	Brain
	SRR1758921	Heart
	SRR1758918	Brain pituitary
	SRR1758917	Brain frontal cortex
	SRR1758916	Brain cerebellum
	SRR1758915	Bone Marrow
**Pan troglodytes**	SRR1602576	Blood
	SRR1758944	Lymph node
	SRR1758961	Thymus
	SRR1758960	Spleen
	SRR1758959	Skeletal muscle
	SRR1758958	Lymph node
	SRR1758957	Lung
	SRR1758956	Liver
	SRR1758955	Kidney
	SRR1758954	Heart
	SRR1758953	Colon
	SRR1758952	Brain temporal lobe
	SRR1758950	Brain frontal cortex
	SRR1758949	Brain cerebellum
	SRR1758948	Brain cerebellum
	SRR1758947	Thymus
	SRR1758946	Spleen
	SRR1758945	Skeletal muscle
	SRR1758943	Lung
	SRR1758942	Liver
**Macaca fascicularis**	SRR1758941	Kidney
	SRR306801	Brain, prefrontal cortex
	ERR218143	lymphoblastoid cell lines
**Gorilla gorilla**	ERR218142	lymphoblastoid cell lines
	SRR3053573	testis tissue
	SRR873629	induced pluripotent stem cells
	SRR873628	induced pluripotent stem cells
	SRR873627	induced pluripotent stem cells
	SRR873626	induced pluripotent stem cells
**Pan paniscus**	SRR306826	induced pluripotent stem cells
	ERR247255	Fibroblast
	ERR247254	Fibroblast
	ERR247257	Fibroblast
	ERR247256	Fibroblast
**Pongo abelii**	ERR247258	Fibroblast
	SRR1759026	Liver
	SRR1759032	Thymus

	SRR1759031	Spleen
	SRR1759030	Skeletal muscle
	SRR1759029	Lymph node
	SRR1759028	Lymph node
	SRR1759027	Lung
	SRR1759025	Kidney
	SRR1759024	Heart
	SRR1759023	Colon
	SRR1759022	Brian pituitary
	SRR1759021	Brain frontal cortex
	SRR1759020	Brain frontal cortex
	SRR1759019	Brain cerebellum
	SRR1759018	Brain cerebellum
	SRR1759017	Bone marrow
	SRR1602585	Blood
	SRR832957	unspecifed
**Cercocebus atys**	SRR832956	unspecifed
	SRR1758914	Thymus
	SRR1758913	Spleen
	SRR1758912	Skeletal muscle
	SRR1758911	Skeletal muscle
	SRR1758910	Lymph node
	SRR1758909	Lung
	SRR1758908	Liver
	SRR1758907	Kidney
	SRR1758906	Heart
	SRR1758905	Colon
	SRR1758904	Brain temporal lobe
	SRR1758903	Brain pituitary
	SRR1758902	Brain frontal cortex
	SRR1758901	Brain cerebellum
	SRR1758900	Bone marrow
	SRR1041117	Spleen
	SRR1041116	Lymph node
	SRR1041115	Liver
	SRR1041114	Kidney
**Papio anubis**	SRR1041113	Heart

A list the publications associated with used SRA accessions.

Run accession	DOI	Publication
**Cercocebus atys**		
SRR1602585	doi: 10.1093/nar/gku1110	Peng X et al., "Tissue-specific transcriptome sequencing analysis expands the non-human primate reference transcriptome resource (NHPRTR).", Nucleic Acids Res, 2015 Jan;43(Database issue):D737-42
SRR1759017	doi: 10.1093/nar/gku1110	Peng X et al., "Tissue-specific transcriptome sequencing analysis expands the non-human primate reference transcriptome resource (NHPRTR).", Nucleic Acids Res, 2015 Jan;43(Database issue):D737-42
SRR1759018	doi: 10.1093/nar/gku1110	Peng X et al., "Tissue-specific transcriptome sequencing analysis expands the non-human primate reference transcriptome resource (NHPRTR).", Nucleic Acids Res, 2015 Jan;43(Database issue):D737-42
SRR1759019	doi: 10.1093/nar/gku1110	Peng X et al., "Tissue-specific transcriptome sequencing analysis expands the non-human primate reference transcriptome resource (NHPRTR).", Nucleic Acids Res, 2015 Jan;43(Database issue):D737-42
SRR1759020	doi: 10.1093/nar/gku1110	Peng X et al., "Tissue-specific transcriptome sequencing analysis expands the non-human primate reference transcriptome resource (NHPRTR).", Nucleic Acids Res, 2015 Jan;43(Database issue):D737-42
SRR1759021	doi: 10.1093/nar/gku1110	Peng X et al., "Tissue-specific transcriptome sequencing analysis expands the non-human primate reference transcriptome resource (NHPRTR).", Nucleic Acids Res, 2015 Jan;43(Database issue):D737-42
SRR1759022	doi: 10.1093/nar/gku1110	Peng X et al., "Tissue-specific transcriptome sequencing analysis expands the non-human primate reference transcriptome resource (NHPRTR).", Nucleic Acids Res, 2015 Jan;43(Database issue):D737-42
SRR1759023	doi: 10.1093/nar/gku1110	Peng X et al., "Tissue-specific transcriptome sequencing analysis expands the non-human primate reference transcriptome resource (NHPRTR).", Nucleic Acids Res, 2015 Jan;43(Database issue):D737-42
SRR1759024	doi: 10.1093/nar/gku1110	Peng X et al., "Tissue-specific transcriptome sequencing analysis expands the non-human primate reference transcriptome resource (NHPRTR).", Nucleic Acids Res, 2015 Jan;43(Database issue):D737-42
SRR1759025	doi: 10.1093/nar/gku1110	Peng X et al., "Tissue-specific transcriptome sequencing analysis expands the non-human primate reference transcriptome resource (NHPRTR).", Nucleic Acids Res, 2015 Jan;43(Database issue):D737-42
SRR1759027	doi: 10.1093/nar/gku1110	Peng X et al., "Tissue-specific transcriptome sequencing analysis expands the non-human primate reference transcriptome resource (NHPRTR).", Nucleic Acids Res, 2015 Jan;43(Database issue):D737-42
SRR1759028	doi: 10.1093/nar/gku1110	Peng X et al., "Tissue-specific transcriptome sequencing analysis expands the non-human primate reference transcriptome resource (NHPRTR).", Nucleic Acids Res, 2015 Jan;43(Database issue):D737-42
SRR1759029	doi: 10.1093/nar/gku1110	Peng X et al., "Tissue-specific transcriptome sequencing analysis expands the non-human primate reference transcriptome resource (NHPRTR).", Nucleic Acids Res, 2015 Jan;43(Database issue):D737-42
SRR1759030	doi: 10.1093/nar/gku1110	Peng X et al., "Tissue-specific transcriptome sequencing analysis expands the non-human primate reference transcriptome resource (NHPRTR).", Nucleic Acids Res, 2015 Jan;43(Database issue):D737-42
SRR1759031	doi: 10.1093/nar/gku1110	Peng X et al., "Tissue-specific transcriptome sequencing analysis expands the non-human primate reference transcriptome resource (NHPRTR).", Nucleic Acids Res, 2015 Jan;43(Database issue):D737-42
**Pan troglodytes**		
SRR1602576	doi: 10.1093/nar/gku1110	Peng X et al., "Tissue-specific transcriptome sequencing analysis expands the non-human primate reference transcriptome resource (NHPRTR).", Nucleic Acids Res, 2015 Jan;43(Database issue):D737-42
SRR1758915	doi: 10.1093/nar/gku1110	Peng X et al., "Tissue-specific transcriptome sequencing analysis expands the non-human primate reference transcriptome resource (NHPRTR).", Nucleic Acids Res, 2015 Jan;43(Database issue):D737-42
SRR1758916	doi: 10.1093/nar/gku1110	Peng X et al., "Tissue-specific transcriptome sequencing analysis expands the non-human primate reference transcriptome resource (NHPRTR).", Nucleic Acids Res, 2015 Jan;43(Database issue):D737-42
SRR1758917	doi: 10.1093/nar/gku1110	Peng X et al., "Tissue-specific transcriptome sequencing analysis expands the non-human primate reference transcriptome resource (NHPRTR).", Nucleic Acids Res, 2015 Jan;43(Database issue):D737-42
SRR1758918	doi: 10.1093/nar/gku1110	Peng X et al., "Tissue-specific transcriptome sequencing analysis expands the non-human primate reference transcriptome resource (NHPRTR).", Nucleic Acids Res, 2015 Jan;43(Database issue):D737-42
SRR1758919	doi: 10.1093/nar/gku1110	Peng X et al., "Tissue-specific transcriptome sequencing analysis expands the non-human primate reference transcriptome resource (NHPRTR).", Nucleic Acids Res, 2015 Jan;43(Database issue):D737-42
SRR1758920	doi: 10.1093/nar/gku1110	Peng X et al., "Tissue-specific transcriptome sequencing analysis expands the non-human primate reference transcriptome resource (NHPRTR).", Nucleic Acids Res, 2015 Jan;43(Database issue):D737-42

SRR1758921	doi: 10.1093/nar/gku1110	Peng X et al., "Tissue-specific transcriptome sequencing analysis expands the non-human primate reference transcriptome resource (NHPRTR).", Nucleic Acids Res, 2015 Jan;43(Database issue):D737-42
SRR1758922	doi: 10.1093/nar/gku1110	Peng X et al., "Tissue-specific transcriptome sequencing analysis expands the non-human primate reference transcriptome resource (NHPRTR).", Nucleic Acids Res, 2015 Jan;43(Database issue):D737-42
SRR1758923	doi: 10.1093/nar/gku1110	Peng X et al., "Tissue-specific transcriptome sequencing analysis expands the non-human primate reference transcriptome resource (NHPRTR).", Nucleic Acids Res, 2015 Jan;43(Database issue):D737-42
SRR1758924	doi: 10.1093/nar/gku1110	Peng X et al., "Tissue-specific transcriptome sequencing analysis expands the non-human primate reference transcriptome resource (NHPRTR).", Nucleic Acids Res, 2015 Jan;43(Database issue):D737-42
SRR1758925	doi: 10.1093/nar/gku1110	Peng X et al., "Tissue-specific transcriptome sequencing analysis expands the non-human primate reference transcriptome resource (NHPRTR).", Nucleic Acids Res, 2015 Jan;43(Database issue):D737-42
SRR1758926	doi: 10.1093/nar/gku1110	Peng X et al., "Tissue-specific transcriptome sequencing analysis expands the non-human primate reference transcriptome resource (NHPRTR).", Nucleic Acids Res, 2015 Jan;43(Database issue):D737-42
SRR1758927	doi: 10.1093/nar/gku1110	Peng X et al., "Tissue-specific transcriptome sequencing analysis expands the non-human primate reference transcriptome resource (NHPRTR).", Nucleic Acids Res, 2015 Jan;43(Database issue):D737-42
SRR1758928	doi: 10.1093/nar/gku1110	Peng X et al., "Tissue-specific transcriptome sequencing analysis expands the non-human primate reference transcriptome resource (NHPRTR).", Nucleic Acids Res, 2015 Jan;43(Database issue):D737-42
SRR1758929	doi: 10.1093/nar/gku1110	Peng X et al., "Tissue-specific transcriptome sequencing analysis expands the non-human primate reference transcriptome resource (NHPRTR).", Nucleic Acids Res, 2015 Jan;43(Database issue):D737-42
SRR1758930	doi: 10.1093/nar/gku1110	Peng X et al., "Tissue-specific transcriptome sequencing analysis expands the non-human primate reference transcriptome resource (NHPRTR).", Nucleic Acids Res, 2015 Jan;43(Database issue):D737-42
SRR1758931	doi: 10.1093/nar/gku1110	Peng X et al., "Tissue-specific transcriptome sequencing analysis expands the non-human primate reference transcriptome resource (NHPRTR).", Nucleic Acids Res, 2015 Jan;43(Database issue):D737-42
SRR1758932	doi: 10.1093/nar/gku1110	Peng X et al., "Tissue-specific transcriptome sequencing analysis expands the non-human primate reference transcriptome resource (NHPRTR).", Nucleic Acids Res, 2015 Jan;43(Database issue):D737-42
SRR1758933	doi: 10.1093/nar/gku1110	Peng X et al., "Tissue-specific transcriptome sequencing analysis expands the non-human primate reference transcriptome resource (NHPRTR).", Nucleic Acids Res, 2015 Jan;43(Database issue):D737-42

Papio anubis

SRR1758900	doi: 10.1093/nar/gku1110	Peng X et al., "Tissue-specific transcriptome sequencing analysis expands the non-human primate reference transcriptome resource (NHPRTR).", Nucleic Acids Res, 2015 Jan;43(Database issue):D737-42
SRR1758901	doi: 10.1093/nar/gku1110	Peng X et al., "Tissue-specific transcriptome sequencing analysis expands the non-human primate reference transcriptome resource (NHPRTR).", Nucleic Acids Res, 2015 Jan;43(Database issue):D737-42
SRR1758902	doi: 10.1093/nar/gku1110	Peng X et al., "Tissue-specific transcriptome sequencing analysis expands the non-human primate reference transcriptome resource (NHPRTR).", Nucleic Acids Res, 2015 Jan;43(Database issue):D737-42
SRR1758903	doi: 10.1093/nar/gku1110	Peng X et al., "Tissue-specific transcriptome sequencing analysis expands the non-human primate reference transcriptome resource (NHPRTR).", Nucleic Acids Res, 2015 Jan;43(Database issue):D737-42
SRR1758904	doi: 10.1093/nar/gku1110	Peng X et al., "Tissue-specific transcriptome sequencing analysis expands the non-human primate reference transcriptome resource (NHPRTR).", Nucleic Acids Res, 2015 Jan;43(Database issue):D737-42
SRR1758905	doi: 10.1093/nar/gku1110	Peng X et al., "Tissue-specific transcriptome sequencing analysis expands the non-human primate reference transcriptome resource (NHPRTR).", Nucleic Acids Res, 2015 Jan;43(Database issue):D737-42
SRR1758906	doi: 10.1093/nar/gku1110	Peng X et al., "Tissue-specific transcriptome sequencing analysis expands the non-human primate reference transcriptome resource (NHPRTR).", Nucleic Acids Res, 2015 Jan;43(Database issue):D737-42
SRR1758907	doi: 10.1093/nar/gku1110	Peng X et al., "Tissue-specific transcriptome sequencing analysis expands the non-human primate reference transcriptome resource (NHPRTR).", Nucleic Acids Res, 2015 Jan;43(Database issue):D737-42
SRR1758908	doi: 10.1093/nar/gku1110	Peng X et al., "Tissue-specific transcriptome sequencing analysis expands the non-human primate reference transcriptome resource (NHPRTR).", Nucleic Acids Res, 2015 Jan;43(Database issue):D737-42
SRR1758909	doi: 10.1093/nar/gku1110	Peng X et al., "Tissue-specific transcriptome sequencing analysis expands the non-human primate reference transcriptome resource (NHPRTR).", Nucleic Acids Res, 2015 Jan;43(Database issue):D737-42
SRR1758910	doi: 10.1093/nar/gku1110	Peng X et al., "Tissue-specific transcriptome sequencing analysis expands the non-human primate reference transcriptome resource (NHPRTR).", Nucleic Acids Res, 2015 Jan;43(Database issue):D737-42
SRR1758911	doi: 10.1093/nar/gku1110	Peng X et al., "Tissue-specific transcriptome sequencing analysis expands the non-human primate reference transcriptome resource (NHPRTR).", Nucleic Acids Res, 2015 Jan;43(Database issue):D737-42

SRR1758912	doi: 10.1093/nar/gku1110	Peng X et al., "Tissue-specific transcriptome sequencing analysis expands the non-human primate reference transcriptome resource (NHPRTR).", Nucleic Acids Res, 2015 Jan;43(Database issue):D737-42
SRR1758913	doi: 10.1093/nar/gku1110	Peng X et al., "Tissue-specific transcriptome sequencing analysis expands the non-human primate reference transcriptome resource (NHPRTR).", Nucleic Acids Res, 2015 Jan;43(Database issue):D737-42
SRR1758914	doi: 10.1093/nar/gku1110	Peng X et al., "Tissue-specific transcriptome sequencing analysis expands the non-human primate reference transcriptome resource (NHPRTR).", Nucleic Acids Res, 2015 Jan;43(Database issue):D737-42

Pan paniscus

SRR306826	doi: 10.1038/nature10532.	Brawand D et al., "The evolution of gene expression levels in mammalian organs.", Nature, 2011 Oct 19;478(7369):343-8
SRR873626	doi: 10.1093/hmg/ddx142.	Herai RH et al., "Evidence of nuclei-encoded spliceosome mediating splicing of mitochondrial RNA.", Hum Mol Genet, 2017 Jul 1;26(13):2472-2479
	DOI: 10.1038/nature19075	Nuttle X et al., "Emergence of a Homo sapiens-specific gene family and chromosome 16p11.2 CNV susceptibility.", Nature, 2016 Aug 11;536(7615):205-9
	doi: 10.1038/nature12686	Marchetto MCN et al., "Differential L1 regulation in pluripotent stem cells of humans and apes.", Nature, 2013 Nov 28;503(7477):525-529
SRR873627	doi: 10.1093/hmg/ddx142.	Herai RH et al., "Evidence of nuclei-encoded spliceosome mediating splicing of mitochondrial RNA.", Hum Mol Genet, 2017 Jul 1;26(13):2472-2479
	DOI: 10.1038/nature19075	Nuttle X et al., "Emergence of a Homo sapiens-specific gene family and chromosome 16p11.2 CNV susceptibility.", Nature, 2016 Aug 11;536(7615):205-9
	doi: 10.1038/nature12686	Marchetto MCN et al., "Differential L1 regulation in pluripotent stem cells of humans and apes.", Nature, 2013 Nov 28;503(7477):525-529
SRR873628	doi: 10.1093/hmg/ddx142.	Herai RH et al., "Evidence of nuclei-encoded spliceosome mediating splicing of mitochondrial RNA.", Hum Mol Genet, 2017 Jul 1;26(13):2472-2479
	DOI: 10.1038/nature19075	Nuttle X et al., "Emergence of a Homo sapiens-specific gene family and chromosome 16p11.2 CNV susceptibility.", Nature, 2016 Aug 11;536(7615):205-9
	doi: 10.1038/nature12686	Marchetto MCN et al., "Differential L1 regulation in pluripotent stem cells of humans and apes.", Nature, 2013 Nov 28;503(7477):525-529
SRR873629	doi: 10.1093/hmg/ddx142.	Herai RH et al., "Evidence of nuclei-encoded spliceosome mediating splicing of mitochondrial RNA.", Hum Mol Genet, 2017 Jul 1;26(13):2472-2479
	DOI: 10.1038/nature19075	Nuttle X et al., "Emergence of a Homo sapiens-specific gene family and chromosome 16p11.2 CNV susceptibility.", Nature, 2016 Aug 11;536(7615):205-9
	doi: 10.1038/nature12686	Marchetto MCN et al., "Differential L1 regulation in pluripotent stem cells of humans and apes.", Nature, 2013 Nov 28;503(7477):525-529

Gorilla gorilla

ERR218142	DOI: 10.1038/nature10842	Scally et al., Nature 2012;483;7388;169-75, DOI: 10.1038/nature10842
ERR218143	DOI: 10.1038/nature10842	Scally et al., Nature 2012;483;7388;169-75, DOI: 10.1038/nature10842
SRR3053573	NA	NA
SRR306801	doi: 10.1038/nature10532.	Brawand D et al., "The evolution of gene expression levels in mammalian organs.", Nature, 2011 Oct 19;478(7369):343-8

Chlorocebus Sabaeus

SRR1032363	doi: 10.1038/ng.3959	Anna J. Jasinska et al., Genetic variation and gene expression across multiple tissues and developmental stages in a non-human primate
SRR1032364	doi: 10.1038/ng.3959	Anna J. Jasinska et al., Genetic variation and gene expression across multiple tissues and developmental stages in a non-human primate
SRR1032365	doi: 10.1038/ng.3959	Anna J. Jasinska et al., Genetic variation and gene expression across multiple tissues and developmental stages in a non-human primate
SRR1032370	doi: 10.1038/ng.3959	Anna J. Jasinska et al., Genetic variation and gene expression across multiple tissues and developmental stages in a non-human primate
SRR1032454	doi: 10.1038/ng.3959	Anna J. Jasinska et al., Genetic variation and gene expression across multiple tissues and developmental stages in a non-human primate
SRR1032466	doi: 10.1038/ng.3959	Anna J. Jasinska et al., Genetic variation and gene expression across multiple tissues and developmental stages in a non-human primate
SRR1032471	doi: 10.1038/ng.3959	Anna J. Jasinska et al., Genetic variation and gene expression across multiple tissues and developmental stages in a non-human primate
SRR1032472	doi: 10.1038/ng.3959	Anna J. Jasinska et al., Genetic variation and gene expression across multiple tissues and developmental stages in a non-human primate
SRR1178505	doi: 10.1038/ng.3959	Anna J. Jasinska et al., Genetic variation and gene expression across multiple tissues and developmental stages in a non-human primate
SRR1178508	doi: 10.1038/ng.3959	Anna J. Jasinska et al., Genetic variation and gene expression across multiple tissues and developmental stages in a non-human primate
SRR1184473	doi: 10.1038/ng.3959	Anna J. Jasinska et al., Genetic variation and gene expression across multiple tissues and developmental stages in a non-human primate
SRR1636350	doi: 10.1038/ng.3959	Anna J. Jasinska et al., Genetic variation and gene expression across multiple tissues and developmental stages in a non-human primate
SRR1636374	doi: 10.1038/ng.3959	Anna J. Jasinska et al., Genetic variation and gene expression across multiple tissues and developmental stages in a non-human primate
SRR1636398	doi: 10.1038/ng.3959	Anna J. Jasinska et al., Genetic variation and gene expression across multiple tissues and developmental stages in a non-human primate

SRR1636420	doi: 10.1038/ng.3959	Anna J. Jasinska et al., Genetic variation and gene expression across multiple tissues and developmental stages in a non-human primate
SRR1636445	doi: 10.1038/ng.3959	Anna J. Jasinska et al., Genetic variation and gene expression across multiple tissues and developmental stages in a non-human primate
SRR1636448	doi: 10.1038/ng.3959	Anna J. Jasinska et al., Genetic variation and gene expression across multiple tissues and developmental stages in a non-human primate
SRR1636484	doi: 10.1038/ng.3959	Anna J. Jasinska et al., Genetic variation and gene expression across multiple tissues and developmental stages in a non-human primate
SRR1636489	doi: 10.1038/ng.3959	Anna J. Jasinska et al., Genetic variation and gene expression across multiple tissues and developmental stages in a non-human primate

Microcebus Murinus

SRR1758989	doi: 10.1093/nar/gku1110	Peng X et al., "Tissue-specific transcriptome sequencing analysis expands the non-human primate reference transcriptome resource (NHPRTR).", Nucleic Acids Res, 2015 Jan;43(Database issue):D737-42
SRR1758990	doi: 10.1093/nar/gku1110	Peng X et al., "Tissue-specific transcriptome sequencing analysis expands the non-human primate reference transcriptome resource (NHPRTR).", Nucleic Acids Res, 2015 Jan;43(Database issue):D737-42
SRR1758991	doi: 10.1093/nar/gku1110	Peng X et al., "Tissue-specific transcriptome sequencing analysis expands the non-human primate reference transcriptome resource (NHPRTR).", Nucleic Acids Res, 2015 Jan;43(Database issue):D737-42
SRR1758992	doi: 10.1093/nar/gku1110	Peng X et al., "Tissue-specific transcriptome sequencing analysis expands the non-human primate reference transcriptome resource (NHPRTR).", Nucleic Acids Res, 2015 Jan;43(Database issue):D737-42
SRR1758993	doi: 10.1093/nar/gku1110	Peng X et al., "Tissue-specific transcriptome sequencing analysis expands the non-human primate reference transcriptome resource (NHPRTR).", Nucleic Acids Res, 2015 Jan;43(Database issue):D737-42
SRR1758994	doi: 10.1093/nar/gku1110	Peng X et al., "Tissue-specific transcriptome sequencing analysis expands the non-human primate reference transcriptome resource (NHPRTR).", Nucleic Acids Res, 2015 Jan;43(Database issue):D737-42
SRR1758995	doi: 10.1093/nar/gku1110	Peng X et al., "Tissue-specific transcriptome sequencing analysis expands the non-human primate reference transcriptome resource (NHPRTR).", Nucleic Acids Res, 2015 Jan;43(Database issue):D737-42
SRR1758996	doi: 10.1093/nar/gku1110	Peng X et al., "Tissue-specific transcriptome sequencing analysis expands the non-human primate reference transcriptome resource (NHPRTR).", Nucleic Acids Res, 2015 Jan;43(Database issue):D737-42
SRR1758997	doi: 10.1093/nar/gku1110	Peng X et al., "Tissue-specific transcriptome sequencing analysis expands the non-human primate reference transcriptome resource (NHPRTR).", Nucleic Acids Res, 2015 Jan;43(Database issue):D737-42
SRR1758998	doi: 10.1093/nar/gku1110	Peng X et al., "Tissue-specific transcriptome sequencing analysis expands the non-human primate reference transcriptome resource (NHPRTR).", Nucleic Acids Res, 2015 Jan;43(Database issue):D737-42
SRR1758999	doi: 10.1093/nar/gku1110	Peng X et al., "Tissue-specific transcriptome sequencing analysis expands the non-human primate reference transcriptome resource (NHPRTR).", Nucleic Acids Res, 2015 Jan;43(Database issue):D737-42
SRR1759000	doi: 10.1093/nar/gku1110	Peng X et al., "Tissue-specific transcriptome sequencing analysis expands the non-human primate reference transcriptome resource (NHPRTR).", Nucleic Acids Res, 2015 Jan;43(Database issue):D737-42
SRR1759001	doi: 10.1093/nar/gku1110	Peng X et al., "Tissue-specific transcriptome sequencing analysis expands the non-human primate reference transcriptome resource (NHPRTR).", Nucleic Acids Res, 2015 Jan;43(Database issue):D737-42
SRR1759002	doi: 10.1093/nar/gku1110	Peng X et al., "Tissue-specific transcriptome sequencing analysis expands the non-human primate reference transcriptome resource (NHPRTR).", Nucleic Acids Res, 2015 Jan;43(Database issue):D737-42
SRR1759003	doi: 10.1093/nar/gku1110	Peng X et al., "Tissue-specific transcriptome sequencing analysis expands the non-human primate reference transcriptome resource (NHPRTR).", Nucleic Acids Res, 2015 Jan;43(Database issue):D737-42
SRR832933	doi: 10.1093/nar/gks1268	Pipes L et al., "The non-human primate reference transcriptome resource (NHPRTR) for comparative functional genomics.", Nucleic Acids Res, 2013 Jan;41(Database issue):D906-14

Macaca fascicularis

SRR1758941	doi: 10.1093/nar/gku1110	Peng X et al., "Tissue-specific transcriptome sequencing analysis expands the non-human primate reference transcriptome resource (NHPRTR).", Nucleic Acids Res, 2015 Jan;43(Database issue):D737-42
SRR1758942	doi: 10.1093/nar/gku1110	Peng X et al., "Tissue-specific transcriptome sequencing analysis expands the non-human primate reference transcriptome resource (NHPRTR).", Nucleic Acids Res, 2015 Jan;43(Database issue):D737-42
SRR1758943	doi: 10.1093/nar/gku1110	Peng X et al., "Tissue-specific transcriptome sequencing analysis expands the non-human primate reference transcriptome resource (NHPRTR).", Nucleic Acids Res, 2015 Jan;43(Database issue):D737-42
SRR1758944	doi: 10.1093/nar/gku1110	Peng X et al., "Tissue-specific transcriptome sequencing analysis expands the non-human primate reference transcriptome resource (NHPRTR).", Nucleic Acids Res, 2015 Jan;43(Database issue):D737-42

SRR1758945	doi: 10.1093/nar/gku1110	Peng X et al., "Tissue-specific transcriptome sequencing analysis expands the non-human primate reference transcriptome resource (NHPRTR).", Nucleic Acids Res, 2015 Jan;43(Database issue):D737-42
SRR1758946	doi: 10.1093/nar/gku1110	Peng X et al., "Tissue-specific transcriptome sequencing analysis expands the non-human primate reference transcriptome resource (NHPRTR).", Nucleic Acids Res, 2015 Jan;43(Database issue):D737-42
SRR1758947	doi: 10.1093/nar/gku1110	Peng X et al., "Tissue-specific transcriptome sequencing analysis expands the non-human primate reference transcriptome resource (NHPRTR).", Nucleic Acids Res, 2015 Jan;43(Database issue):D737-42
SRR1758948	doi: 10.1093/nar/gku1110	Peng X et al., "Tissue-specific transcriptome sequencing analysis expands the non-human primate reference transcriptome resource (NHPRTR).", Nucleic Acids Res, 2015 Jan;43(Database issue):D737-42
SRR1758949	doi: 10.1093/nar/gku1110	Peng X et al., "Tissue-specific transcriptome sequencing analysis expands the non-human primate reference transcriptome resource (NHPRTR).", Nucleic Acids Res, 2015 Jan;43(Database issue):D737-42
SRR1758950	doi: 10.1093/nar/gku1110	Peng X et al., "Tissue-specific transcriptome sequencing analysis expands the non-human primate reference transcriptome resource (NHPRTR).", Nucleic Acids Res, 2015 Jan;43(Database issue):D737-42
SRR1758952	doi: 10.1093/nar/gku1110	Peng X et al., "Tissue-specific transcriptome sequencing analysis expands the non-human primate reference transcriptome resource (NHPRTR).", Nucleic Acids Res, 2015 Jan;43(Database issue):D737-42
SRR1758953	doi: 10.1093/nar/gku1110	Peng X et al., "Tissue-specific transcriptome sequencing analysis expands the non-human primate reference transcriptome resource (NHPRTR).", Nucleic Acids Res, 2015 Jan;43(Database issue):D737-42
SRR1758954	doi: 10.1093/nar/gku1110	Peng X et al., "Tissue-specific transcriptome sequencing analysis expands the non-human primate reference transcriptome resource (NHPRTR).", Nucleic Acids Res, 2015 Jan;43(Database issue):D737-42
SRR1758955	doi: 10.1093/nar/gku1110	Peng X et al., "Tissue-specific transcriptome sequencing analysis expands the non-human primate reference transcriptome resource (NHPRTR).", Nucleic Acids Res, 2015 Jan;43(Database issue):D737-42
SRR1758956	doi: 10.1093/nar/gku1110	Peng X et al., "Tissue-specific transcriptome sequencing analysis expands the non-human primate reference transcriptome resource (NHPRTR).", Nucleic Acids Res, 2015 Jan;43(Database issue):D737-42
SRR1758957	doi: 10.1093/nar/gku1110	Peng X et al., "Tissue-specific transcriptome sequencing analysis expands the non-human primate reference transcriptome resource (NHPRTR).", Nucleic Acids Res, 2015 Jan;43(Database issue):D737-42
SRR1758958	doi: 10.1093/nar/gku1110	Peng X et al., "Tissue-specific transcriptome sequencing analysis expands the non-human primate reference transcriptome resource (NHPRTR).", Nucleic Acids Res, 2015 Jan;43(Database issue):D737-42
SRR1758959	doi: 10.1093/nar/gku1110	Peng X et al., "Tissue-specific transcriptome sequencing analysis expands the non-human primate reference transcriptome resource (NHPRTR).", Nucleic Acids Res, 2015 Jan;43(Database issue):D737-42
SRR1758960	doi: 10.1093/nar/gku1110	Peng X et al., "Tissue-specific transcriptome sequencing analysis expands the non-human primate reference transcriptome resource (NHPRTR).", Nucleic Acids Res, 2015 Jan;43(Database issue):D737-42
SRR1758961	doi: 10.1093/nar/gku1110	Peng X et al., "Tissue-specific transcriptome sequencing analysis expands the non-human primate reference transcriptome resource (NHPRTR).", Nucleic Acids Res, 2015 Jan;43(Database issue):D737-42

Callithrix jacchus

ERR731511		This data is part of a pre-publication release.
SRR1758977	doi: 10.1093/nar/gku1110	Peng X et al., "Tissue-specific transcriptome sequencing analysis expands the non-human primate reference transcriptome resource (NHPRTR).", Nucleic Acids Res, 2015 Jan;43(Database issue):D737-42
SRR1758978	doi: 10.1093/nar/gku1110	Peng X et al., "Tissue-specific transcriptome sequencing analysis expands the non-human primate reference transcriptome resource (NHPRTR).", Nucleic Acids Res, 2015 Jan;43(Database issue):D737-42
SRR1758979	doi: 10.1093/nar/gku1110	Peng X et al., "Tissue-specific transcriptome sequencing analysis expands the non-human primate reference transcriptome resource (NHPRTR).", Nucleic Acids Res, 2015 Jan;43(Database issue):D737-42
SRR1758980	doi: 10.1093/nar/gku1110	Peng X et al., "Tissue-specific transcriptome sequencing analysis expands the non-human primate reference transcriptome resource (NHPRTR).", Nucleic Acids Res, 2015 Jan;43(Database issue):D737-42
SRR1758982	doi: 10.1093/nar/gku1110	Peng X et al., "Tissue-specific transcriptome sequencing analysis expands the non-human primate reference transcriptome resource (NHPRTR).", Nucleic Acids Res, 2015 Jan;43(Database issue):D737-42
SRR1758983	doi: 10.1093/nar/gku1110	Peng X et al., "Tissue-specific transcriptome sequencing analysis expands the non-human primate reference transcriptome resource (NHPRTR).", Nucleic Acids Res, 2015 Jan;43(Database issue):D737-42
SRR1758984	doi: 10.1093/nar/gku1110	Peng X et al., "Tissue-specific transcriptome sequencing analysis expands the non-human primate reference transcriptome resource (NHPRTR).", Nucleic Acids Res, 2015 Jan;43(Database issue):D737-42
SRR1758985	doi: 10.1093/nar/gku1110	Peng X et al., "Tissue-specific transcriptome sequencing analysis expands the non-human primate reference transcriptome resource (NHPRTR).", Nucleic Acids Res, 2015 Jan;43(Database issue):D737-42

SRR1758986	doi: 10.1093/nar/gku1110	Peng X et al., "Tissue-specific transcriptome sequencing analysis expands the non-human primate reference transcriptome resource (NHPRTR).", Nucleic Acids Res, 2015 Jan;43(Database issue):D737-42
SRR1758987	doi: 10.1093/nar/gku1110	Peng X et al., "Tissue-specific transcriptome sequencing analysis expands the non-human primate reference transcriptome resource (NHPRTR).", Nucleic Acids Res, 2015 Jan;43(Database issue):D737-42
SRR1758988	doi: 10.1093/nar/gku1110	Peng X et al., "Tissue-specific transcriptome sequencing analysis expands the non-human primate reference transcriptome resource (NHPRTR).", Nucleic Acids Res, 2015 Jan;43(Database issue):D737-42
SRR629515	doi: 10.1093/nar/gkr1163	BioProject and BioSample databases at NCBI: facilitating capture and organization of metadata
SRR629519	doi: 10.1093/nar/gkr1163	BioProject and BioSample databases at NCBI: facilitating capture and organization of metadata
SRR866208	doi: 10.1186/2047-217X-3-14	Maudhoo MD et al., "De novo assembly of the common marmoset transcriptome from NextGen mRNA sequences.", Gigascience, 2014;3:14
SRR866209	doi: 10.1186/2047-217X-3-14	Maudhoo MD et al., "De novo assembly of the common marmoset transcriptome from NextGen mRNA sequences.", Gigascience, 2014;3:14
SRR867043	doi: 10.1186/2047-217X-3-14	Maudhoo MD et al., "De novo assembly of the common marmoset transcriptome from NextGen mRNA sequences.", Gigascience, 2014;3:14
SRR867044	doi: 10.1186/2047-217X-3-14	Maudhoo MD et al., "De novo assembly of the common marmoset transcriptome from NextGen mRNA sequences.", Gigascience, 2014;3:14
SRR952610	NA	NA

Pongo abelii

ERR247254	NA	NA
ERR247255	NA	NA
ERR247256	NA	NA
ERR247257	NA	NA
ERR247258	NA	NA

Macaca mulatta – Rhesus

ERR247244		
SRR1048277	doi: 10.1093/dnares/dsu019	Zhao Y et al., "mRNA-Seq and microRNA-Seq whole-transcriptome analyses of rhesus monkey embryonic stem cell neural differentiation revealed the potential regulators of rosette neural stem cells.", DNA Res, 2014 Oct;21(5):541-54
SRR1240160	doi: 10.1128/JVI.00543-14.	Barrenas F et al., "Deep transcriptional sequencing of mucosal challenge compartment from rhesus macaques acutely infected with simian immunodeficiency virus implicates loss of cell adhesion preceding immune activation.", J Virol, 2014 Jul;88(14):7962-72
SRR1282462	NA	NA
SRR1282481	NA	NA
SRR1369674	doi: 10.3389/fcell.2014.00054.	Lee KJ et al., "Comparative transcriptomics and metabolomics in a rhesus macaque drug administration study.", Front Cell Dev Biol, 2014;2:54
SRR1575130	doi: 10.1093/nar/gku1110.	Peng X et al., "Tissue-specific transcriptome sequencing analysis expands the non-human primate reference transcriptome resource (NHPRTR).", Nucleic Acids Res, 2015 Jan;43(Database issue):D737-42
SRR1602582	doi: 10.1093/nar/gku1110.	Peng X et al., "Tissue-specific transcriptome sequencing analysis expands the non-human primate reference transcriptome resource (NHPRTR).", Nucleic Acids Res, 2015 Jan;43(Database issue):D737-42
SRR1746861		
SRR1778441	doi: 10.1093/molbev/msv183	Yang XZ et al., "Selectively Constrained RNA Editing Regulation Crosstalks with piRNA Biogenesis in Primates.", Mol Biol Evol, 2015 Dec;32(12):3143-57
	doi: 10.1371/journal.pgen.1004274.	Chen JY et al., "RNA editome in rhesus macaque shaped by purifying selection.", PLoS Genet, 2014 Apr;10(4):e1004274
SRR223522		Yan G et al., "Genome sequencing and comparison of two nonhuman primate animal models, the cynomolgus and Chinese rhesus macaques.", Nat Biotechnol, 2011 Oct 16;29(11):1019-23
SRR389099	doi: 10.1093/molbev/msv183	Yang XZ et al., "Selectively Constrained RNA Editing Regulation Crosstalks with piRNA Biogenesis in Primates.", Mol Biol Evol, 2015 Dec;32(12):3143-57
	doi: 10.1371/journal.pgen.1004274	Chen JY et al., "RNA editome in rhesus macaque shaped by purifying selection.", PLoS Genet, 2014 Apr;10(4):e1004274
	doi: 10.1093/nar/gks835	Zhang SJ et al., "RhesusBase: a knowledgebase for the monkey research community.", Nucleic Acids Res, 2013 Jan;41(Database issue):D892-905
	doi: 10.1371/journal.pgen.1002942	Xie C et al., "Hominoid-specific de novo protein-coding genes originating from long non-coding RNAs.", PLoS Genet, 2012 Sep;8(9):e1002942
SRR389100	doi: 10.1093/molbev/msv183	Yang XZ et al., "Selectively Constrained RNA Editing Regulation Crosstalks with piRNA Biogenesis in Primates.", Mol Biol Evol, 2015 Dec;32(12):3143-57
	doi: 10.1371/journal.pgen.1004274	Chen JY et al., "RNA editome in rhesus macaque shaped by purifying selection.", PLoS Genet, 2014 Apr;10(4):e1004274
	doi: 10.1093/nar/gks835	Zhang SJ et al., "RhesusBase: a knowledgebase for the monkey research community.", Nucleic Acids Res, 2013 Jan;41(Database issue):D892-905
	doi: 10.1371/journal.pgen.1002942	Xie C et al., "Hominoid-specific de novo protein-coding genes originating from long non-coding RNAs.", PLoS Genet, 2012 Sep;8(9):e1002942
SRR389102	doi: 10.1093/molbev/msv183	Yang XZ et al., "Selectively Constrained RNA Editing Regulation Crosstalks with piRNA Biogenesis in Primates.", Mol Biol Evol, 2015 Dec;32(12):3143-57

	doi: 10.1371/journal.pgen.1004274	Chen JY et al., "RNA editome in rhesus macaque shaped by purifying selection.", PLoS Genet, 2014 Apr;10(4):e1004274
	doi: 10.1093/nar/gks835	Zhang SJ et al., "RhesusBase: a knowledgebase for the monkey research community.", Nucleic Acids Res, 2013 Jan;41(Database issue):D892-905
	doi: 10.1371/journal.pgen.1002942	Xie C et al., "Hominoid-specific de novo protein-coding genes originating from long non-coding RNAs.", PLoS Genet, 2012 Sep;8(9):e1002942
SRR594458	doi: 10.1126/science.1228186	Merkin J et al., "Evolutionary dynamics of gene and isoform regulation in Mammalian tissues.", Science, 2012 Dec 21;338(6114):1593-9
SRR594460	doi: 10.1126/science.1228186	Merkin J et al., "Evolutionary dynamics of gene and isoform regulation in Mammalian tissues.", Science, 2012 Dec 21;338(6114):1593-9
SRR594462	doi: 10.1126/science.1228186	Merkin J et al., "Evolutionary dynamics of gene and isoform regulation in Mammalian tissues.", Science, 2012 Dec 21;338(6114):1593-9

Suppl. File 2. A summary of the non-human primate species used in the research.

№	Name	Full species name	Genome version	lncRNAs
1	Chimp	*Pan troglodytes*	CHIMP2.1.4	18227
2	Bonobo	*Pan paniscus*	PanPan1.1	2055
3	Gorilla	*Gorilla gorilla*	gorGor3.1.83	9965
4	Orangutan	*Pongo Abelii*	PPYG2.86	3113
5	Rhesus	*Macaca Mulatta*	MMUL_1.83	8435
6	Macaca Fascicularis	*Macaca fascicularis*	MacFas5	16726
7	Atys	*Cercocebus atys*	Caty 1.0	11634
8	Baboon	*Papio anubis*	PapAnu2.0.84	13608
9	GreenMonkey	*Chlorocebus sabaeus*	Chlorocebus_sabeus 1.1 / chlSab2	10267
10	Marmoset	*Callithrix jacchus*	C_jacchus3.2.1.83.	10853
11	Lemur	*Microcebus_murinus*	micMur1.83	11416

№	Name	Full species name	Used GTF/GFF
1	Chimp	*Pan troglodytes*	Pan_troglodytes.CHIMP2.1.4.84.gtf
2	Bonobo	*Pan paniscus*	GCF_000258655.2_panpan1.1_genomic.gff
3	Gorilla	*Gorilla gorilla*	Gorilla_gorilla.gorGor3.1.83.gtf
4	Orangutan	*Pongo Abelii*	Pongo_abelii.PPYG2.84.gtf
5	Rhesus	*Macaca Mulatta*	Macaca_mulatta.MMUL_1.83.gtf
6	Macaca Fascicularis	*Macaca fascicularis*	GCF_000364345.1_Macaca_fascicularis_5.0_genomic.gff
7	Atys	*Cercocebus atys*	ref_Chlorocebus_sabeus_1.1_top_level.gff3
8	Baboon	*Papio anubis*	Papio_anubis.PapAnu2.0.84.gtf
9	GreenMonkey	*Chlorocebus sabaeus*	ref_Chlorocebus_sabeus_1.1_top_level.gff3
10	Marmoset	*Callithrix jacchus*	Callithrix_jacchus.C_jacchus3.2.1.83.gtf
11	Lemur	*Microcebus_murinus*	Microcebus_murinus.micMur1.83.gtf

Suppl. File 3

A list of Cuffcompare class codes with their meanings.

Code	Description
=	Complete match of intron chain
c	Contained
j	Potentially novel isoform (fragment): at least one splice junction is shared with a reference transcript
e	Single exon transfrag overlapping a reference exon and at least 10 bp of a reference intron, indicating a possible pre-mRNA fragment.
i	A transfrag falling entirely within a reference intron
o	Generic exonic overlap with a reference transcript
p	Possible polymerase run-on fragment (within 2Kbases of a reference transcript)
r	Repeat. Currently determined by looking at the soft-masked reference sequence and applied to transcripts where at least 50% of the bases are lower case
u	Unknown, intergenic transcript
x	Exonic overlap with reference on the opposite strand
s	An intron of the transfrag overlaps a reference intron on the opposite strand (likely due to read mapping errors)
.	(.tracking file only, indicates multiple classifications)
